# A corn-fermented protein ingredient can be included in early nursey diets without compromising pig growth performance and health status

**DOI:** 10.1093/tas/txad149

**Published:** 2024-01-05

**Authors:** Yesid R Garavito-Duarte, Crystal L Levesque, Kevin Herrick, Jorge Y Perez-Palencia

**Affiliations:** Department of Animal Science, South Dakota State University, Brookings, SD 57007, USA; Department of Animal Science, North Carolina State University, Raleigh, NC 27695, USA; Department of Animal Science, South Dakota State University, Brookings, SD 57007, USA; POET Nutrition, Sioux Falls, SD 57104, USA; Department of Animal Science, South Dakota State University, Brookings, SD 57007, USA

**Keywords:** intestinal health, nursery diets, nutrient digestibility, protein source, weaned pig, yeast

## Abstract

In nursery diets, ingredients with high protein content and highly digestible nutrients, such as corn-fermented protein product with added yeast mass (**GDDY**), can be included as an alternative to common protein sources. This study investigated the dietary inclusion of GDDY as an alternative protein source on growth performance and intestinal health of weaned pigs. A total of 594 weaned pigs (5.7 ± 0.9 kg; 18.5 days of age) were allotted to 36 pens in a randomized incomplete block design. Pens were assigned to one of 4 dietary treatments: **CON**: a common nursery feeding program; **SBM75**: CON diet replacing 75% of soybean meal (**SBM**) with GDDY; **FM/ESBM**: CON diet without fish meal (**FM**) and enzyme-treated SBM (**ESBM**) + GDDY; **GDDY50**: CON diet replacing 50% of SBM, FM, and ESBM with GDDY. Experimental diets were formulated to meet nutrient requirements of nursery pigs and provided in meal form through four phases during the nursery period. Pig growth performance was assessed on days 7, 14, 21, 28, 42, and 53. Pen fecal score was assessed daily from days 0 to 14, and 3 times per week from days 15 to 35. Intestinal health was assessed based on plasma immunoglobulin A (**IgA**) concentration and the differential sugar absorption test. The total tract digestibility of dry matter (**DM**), crude protein (**CP**), gross energy (**GE**), and phosphorus was also evaluated. From days 0 to 7 and days 7 to 14, dietary treatment had no effect (*P* > 0.05) on BW, ADG, and ADFI. For the rest of the experimental period, ADG and ADFI were greater (*P* < 0.05) in pigs fed CON in comparison with those fed SBM75 and GDDY50 and did not differ from pigs fed FM/ESBM. Pigs fed GDDY50 tended (*P* = 0.082) to have greater serum IgA concentration on day 20 when compared with SBM75 and FM/ESBM pigs. There were no differences among dietary treatments for DM, CP, and GE digestibility. Phosphorus digestibility was higher in FM/ESBM (*P* < 0.05) compared with SBM75 and GDDY50. These results supported the hypothesis that GDDY can be incorporated in nursery pig diets during the first couple weeks after weaning without affecting growth performance. However, in the late nursery period, inclusion levels starting at 14% can compromise performance.

## INTRODUCTION

The weaning phase is one of the most critical periods during pig production due to the combined stressors faced by piglets such as new environment, social interactions, and change of diet (Zheng et al. 2021). All these stressors lead to increased susceptibility to gut disorders, which, in concert with immature digestive and immune system, results in increased incidences of diarrhea, poor growth rate, increased mortality and (or) morbidity, and subsequent economic losses for swine producers (Lallès et al. 2004, Smith et al. 2010, Pohl et al. 2017). Therefore, it is important to implement different management strategies to reduce the stress associated with weaning and promote the physiological development of pigs. One of the commonly used strategies during this period is related to diet formulation, especially the use of ingredients with both high protein content and digestibility.

The formulation of diets to reduce the negative effects of weaning using ingredients with high digestibility and palatability typically increases diet cost. Fishmeal (**FM**), spray-dried plasma, enzyme-treated SBM (**ESBM**), products derived from blood, and even cereals processed with thermal methods are included as high-quality protein sources in diets for nursery pigs commonly referred to as “complex” diets (Collins et al. 2017). Although feeding pigs with diets rich in high-quality protein during the weaning phase are considered essential to guarantee optimal growth in the transition and later phases, the high price and low availability of these ingredients increases the overall cost of pork production (Totafurno et al. 2019). On the other hand, soybean meal (**SBM**) is the most common protein source used in swine diets because of its high protein and amino acid (**AA**) content and consistent quality and availability in the market (Chiba 2013). However, its inclusion as a feed ingredient is limited, particularly in young animal diets, due to relatively high levels of antinutritional factors (**ANFs**) and nonstarch polysaccharides (**NSPs**). The presence of ANFs such as trypsin inhibitors, protease inhibitors, lectins, phytoestrogens, oligosaccharides, and phytate interferes with digestion, absorption, and nutrient utilization that can lower overall nutritional value of SBM and negatively affect pig growth performance (Liener 1994, Pettersson and Pontoppidan 2013, Jeong et al. 2016). In addition, two major proteins in SBM (glycinin and β-conglycinin) induce allergic reaction and have been associated with inflammatory response in young pigs (Fu et al. 2007, Song et al. 2010) Thus, the inclusion of SBM in nursery diets is typically restricted (<20%) and gradually increased through their subsequent growth periods (Stein et al. 2013). In this context, it is important to find alternative protein sources that are cost-effective in nursery pig diets without compromising pig performance and health.

Corn-fermented protein products have been developed through separation of the protein fraction from distiller’s dried grains with solubles (**DDGS**) and are expected to contain greater concentrations of yeast which may contribute to a better AA profile. In our previous study, corn-fermented protein products with added yeast mass (**GDDY**) had similar standardized ileal digestibility of AA when compared with SBM and FM (Garavito-Duarte et al. 2023). In addition, the metabolizable energy content was comparable to corn, one of the main energy sources used in commercial pig diets in the United States. Therefore, the objective of this study was to evaluate the inclusion of GDDY as an alternative protein resource in nursery diets on pig growth performance and intestinal barrier function.

## MATERIALS AND METHODS

This study was approved by the Institutional Animal Care and Use Committee at the South Dakota State University and Use Committee (IACUC # 2105-023A).

### Animals and Experimental Design

A total of 594 barrows and gilts (5.7 ± 0.9 kg; 18.5 days of age) were used in a randomized incomplete block design at the SDSU Swine Research and Education Center. Pigs were weaned into 36 pens (15 to 18 pigs per pen) in 2 separate room (18 pens per room), sex ratios were maintained within 4 body weight (BW) groups and each treatment was equally represented within each BW group. The average BW in each block was 4.32 kg ± 0.340, 5.28 kg ± 0.177, 5.99 kg ± 0.385, and 6.84 kg ± 0.451, respectively. The facility operated with mechanical ventilation, with room temperature set at 30, 29, 28, 26.5, 25, 24 °C during weeks 1 to 6 of the nursery period.

The experimental diets (Tables 1 and 2) were formulated to meet or exceed nutrient requirements for weaned pigs (NRC 2012, except Met + Cys) and provided in meal form through 4 phases during the nursery period (42 days): Phase 1 (days 0 to 7), Phase 2 (days 7 to 21), Phase 3 (days 21 to 28) and Phase 4 (days 28 to 53). Pens were assigned to one of 4 dietary treatments resulting in 9 pens per treatment. The dietary treatments were **CON**: a common nursery feeding program; SBM75: CON diet replacing 75% of SBM with GDDY; **FM/ESBM**: CON diet without FM and ESBM + GDDY inclusion; **GDDY50**: CON diet replacing 50% of SBM, FM, and ESBM by GDDY. The GDDY was obtained by separating the protein fraction of DDGS and adding yeast mass derived from the fermentation process during the ethanol production, then drying the stillage and fermented residues by the ring dryer method. The final product contained 50.01% CP content, Lys 2.22%, Ile 2.32%, Leu 5.24%, Met 1.10%, Thr 2.04%, Trp 0.54%, and Val 2.99%. Additional nutrient details are available in Garavito-Duarte et al., (2023).

**Table 1.** Formulation and calculated composition of experimental diets for phase 1 (days 0 to 7) and phase 2 (days 7 to 21), as-fed basis^1^

Ingredient	Phase 1				Phase 2			
	CON	SBM75	FM/ESBM	GDDY50	CON	SBM75	FM/ESBM	GDDY50
Corn	38.31	38.10	33.08	36.99	43.59	42.87	39.64	42.49
Soybean meal	18.00	4.00	18.00	9.00	20.00	5.00	20.00	10.00
Dried Whey	30.00	30.00	30.00	30.00	25.00	25.00	25.00	25.00
Fish meal	5.00	5.00	—	2.50	4.00	4.00	—	2.00
HP 300^2^	5.00	5.00	—	2.50	4.00	4.00	—	2.00
Ring GDDY^3^	0.00	13.50	14.00	14.00	0.00	15.00	11.00	14.00
l-Lysine HCl	0.46	0.64	0.60	0.68	0.33	0.51	0.44	0.54
l-Threonine	0.17	0.16	0.16	0.18	0.11	0.09	0.10	0.11
dl-Methionine	0.12	0.07	0.09	0.09	0.09	0.03	0.07	0.05
l-Tryptophan	0.00	0.03	—	0.03	—	0.02	—	0.02
l-Valine	0.07	—	—	0.02	—	—	—	—
Soybean oil	1.00	1.11	1.15	1.29	0.80	0.85	0.88	0.98
Monocalcium phosphate	0.15	—	0.42	0.17	0.14	0.00	0.36	0.10
Limestone	0.95	1.10	1.42	1.30	1.03	1.16	1.38	1.32
Potassium chloride	—	0.52	0.24	0.45	0.00	0.56	0.18	0.46
Salt	0.15	0.15	0.22	0.18	0.16	0.16	0.20	0.18
Swine vitamin premix^4^	0.05	0.05	0.05	0.05	0.05	0.05	0.05	0.05
Swine mineral premix^5^	0.15	0.15	0.15	0.15	0.15	0.15	0.15	0.15
Zinc oxide	0.42	0.42	0.42	0.42	0.42	0.42	0.42	0.42
Swine larvicide	0.13	0.13	0.13	0.13	0.13	0.13	0.13	0.13
*Calculated composition*								
Metabolizable energy, kcal/kg	3,521	3,521	3,521	3,521	3,503	3,503	3,503	3,498
Crude protein, %	21.5	21.5	21.9	21.1	21.0	21.0	21.0	21.0
Lactose	22.0	22.0	22.0	22.0	18.0	18.0	18.0	18.0
*Standardized ileal digestible AA*								
Lys, %	1.50	1.50	1.50	1.50	1.35	1.35	1.35	1.35
Met, %	0.43	0.43	0.43	0.43	0.39	0.39	0.39	0.39
Met + Cys, %	0.71	0.72	0.74	0.72	0.67	0.68	0.69	0.68
Thr, %	0.88	0.88	0.88	0.88	0.79	0.79	0.78	0.79
Trp, %	0.25	0.25	0.25	0.25	0.24	0.24	0.24	0.24
Ile, %	0.85	0.84	0.89	0.83	0.83	0.82	0.86	0.81
Leu, %	1.56	1.76	1.81	1.74	1.54	1.77	1.73	1.73
Val, %	0.95	0.95	0.97	0.95	0.86	0.94	0.92	0.91
His, %	0.46	0.47	0.49	0.46	0.46	0.47	0.48	0.46
Phe, %	0.83	0.86	0.91	0.85	0.82	0.87	0.89	0.85
Calcium, %	0.85	0.85	0.85	0.85	0.80	0.80	0.80	0.80
Phosphorus, %	0.65	0.65	0.64	0.63	0.60	0.61	0.60	0.58
ATTD P, %	0.41	0.43	0.41	0.41	0.36	0.39	0.36	0.36
STTD P, %	0.45	0.48	0.46	0.46	0.40	0.43	0.41	0.41

^1^Dietary treatments: CON: Standard nursery diet; SBM75: CON replacing 75% of SBM by GDDY; FM/ESBM: CON without Fishmeal and ESBM + GDDY inclusion; HP-GDDY50: CON replacing 50% of SBM, FM, and ESBM by GDDY; AA: amino acids; ATTD: apparent total tract digestibility; P: phosphorus; STTD: standardized total tract digestibility.

^2^Enzymatic treated soybean meal, HAMLET PROTEIN Inc., Findlay, OH.

^3^Ring GDDY, corn-fermented protein product. POET, LLC, Sioux Falls, SD.

^4^J & R Distributing Inc. 518 Main Avenue, Lake Norden, SD 57248, . Minimum provided per kg of diet: calcium 55 mg, vitamin A 11,000 IU, vitamin D3 1,650 IU, vitamin E 55 IU; vitamin B12 0.044 mg, menadione 4.4 mg, biotin 0.165 mg, folic acid 1.1 mg, niacin 55 mg, d-pantothenic acid 60.5 mg, vitamin B16 3.3 mg, riboflavin 9.9 mg, thiamine 3.3 mg.

^5^J & R Distributing Inc. 518 Main Avenue, Lake Norden, SD 57248, . Minimum provided per kg of diet: copper 16.5 ppm, manganese 44.1 ppm, selenium 0.3 ppm, zinc 165 ppm.

**Table 2.** Formulation and calculated composition of experimental diets for phase 3 (days 21 to 28) and phase 4 (days 28 to 53), as-fed basis^1^

Ingredient	Phase 3				Phase 4			
	CON	SBM75	FM/ESBM	GDDY50	CON	SBM75	FM/ESBM	GDDY50
Corn	53.08	52.32	50.53	52.11	65.80	64.90	65.80	65.18
Soybean meal	28.00	7.00	28.00	14.00	30.00	7.50	30.00	15.00
Dried Whey	12.00	12.00	12.00	12.00	—	—	—	—
Fish meal	3.00	3.00	—	1.50	—	—	—	—
HP 300^2^	—	—	—	—	—	—	—	—
Ring GDDY^3^	—	21.00	5.00	15.50	—	22.50	—	15.00
l-Lysine HCl	0.38	0.63	0.42	0.58	0.40	0.67	0.40	0.58
l-Threonine	0.16	0.13	0.15	0.15	0.18	0.14	0.18	0.16
dl-Methionine	0.09	0.01	0.09	0.04	0.09	—	0.09	0.03
l-Tryptophan	—	0.03	—	0.02	—	0.03	—	0.02
l-Valine	0.02	—	—	—	—	—	—	—
Soybean oil	0.50	0.48	0.55	0.63	0.50	0.50	0.50	0.50
Monocalcium phosphate	0.60	0.18	0.82	0.46	0.90	0.45	0.90	0.63
Limestone	1.10	1.40	1.32	1.40	1.20	1.55	1.20	1.42
Potassium chloride	—	0.76	0.01	0.52	—	0.83	—	0.55
Salt	0.46	0.45	0.50	0.48	0.60	0.60	0.60	0.60
Swine vitamin premix^4^	0.05	0.05	0.05	0.05	0.05	0.05	0.05	0.05
Swine mineral premix ^5^	0.15	0.15	0.15	0.15	0.15	0.15	0.15	0.15
Zinc oxide	0.28	0.28	0.28	0.28	—	—	—	—
Swine Larvicide	0.13	0.13	0.13	0.13	0.13	0.13	0.13	0.13
*Calculated composition*								
Metabolizable energy, kcal/kg	3,474	3,474	3,474	3,474	3,458	3,458	3,458	3,458
Crude Protein, %	21.2	21.5	21.5	21.1	21.1	21	20.1	20.1
Lactose	9	9	9	9	0	0	0	0
*Standardized ileal digestible AA*								
Lys, %	1.35	1.35	1.35	1.35	1.23	1.23	1.23	1.23
Met, %	0.39	0.39	0.39	0.39	0.36	0.36	0.36	0.36
Met + Cys, %	0.66	0.68	0.68	0.68	0.62	0.64	0.63	0.63
Thr, %	0.79	0.79	0.79	0.79	0.73	0.72	0.73	0.73
Trp, %	0.23	0.23	0.24	0.23	0.21	0.21	0.21	0.21
Ile, %	0.81	0.81	0.84	0.81	0.75	0.74	0.74	0.74
Leu, %	1.54	1.86	1.64	1.77	1.47	1.82	1.70	1.70
Val, %	0.86	0.95	0.89	0.92	0.78	0.90	0.86	0.86
His, %	0.48	0.49	0.49	0.48	0.46	0.48	0.47	0.47
Phe, %	0.85	0.92	0.91	0.90	0.84	0.91	0.89	0.89
Calcium, %	0.80	0.80	0.80	0.80	0.70	0.70	0.70	0.70
Phosphorus, %	0.63	0.60	0.63	0.61	0.56	0.53	0.55	0.55
ATTD P, %	0.36	0.36	0.36	0.36	0.29	0.29	0.29	0.29
STTD P, %	0.40	0.40	0.41	0.40	0.33	0.33	0.33	0.33

^1^Dietary treatments: CON: standard nursery diet; SBM75: CON replacing 75% of SBM by GDDY; FM/ESBM: CON without fishmeal and ESBM + GDDY inclusion; HP-GDDY50: CON replacing 50% of SBM, FM, and ESBM by GDDY; AA: amino acids; ATTD: apparent total tract digestibility; P: phosphorus; STTD: standardized total tract digestibility.

^2^Enzymatic treated soybean meal, HAMLET PROTEIN Inc., Findlay, OH.

^3^Ring GDDY, corn-fermented protein product. POET, LLC, Sioux Falls, SD.

^4^J & R Distributing Inc. 518 Main Avenue, Lake Norden, SD,. Minimum provided per kg of diet: Calcium 55 mg, vitamin A 11,000 IU, vitamin D3 1,650 IU, vitamin E 55 IU; vitamin B12 0.044 mg, menadione 4.4 mg, biotin 0.165 mg, folic acid 1.1 mg, niacin 55 mg, d-pantothenic acid 60.5 mg, vitamin B16 3.3 mg, riboflavin 9.9 mg, thiamine 3.3 mg.

^5^J & R Distributing Inc., Lake Norden, SD. Minimum provided per kg of diet: copper 16.5 ppm, manganese 44.1 ppm, selenium 0.3 ppm, zinc 165 ppm.

### Experimental Procedures

Daily animal care observations included pig behavior, daily room temperature recording, check waterers and feeders, and treatment of pigs when necessary. Pigs were treated when they showed clinical signs of disease. Treatment dose, product used, date of administration, identification of pig and pen, and reason for treatment were recorded throughout the experimental period.

### Growth Performance

Pigs were weighed on days 0, 7, 14, 21, 28, 42, and 53. Feed disappearance was measured simultaneously with body weight (**BW**), and average daily gain (**ADG**), average daily feed intake (**ADFI**), and gain:feed (**G:F**) were calculated.

### Fecal Score and Intestinal Health

Daily from days 0 to 14 and 3 times a week from days 15 to 35, pen fecal score was visually assessed using a 4-category fecal consistency scale (Pedersen and Toft 2011). The 4 categories were score 1 = firm and shaped, score 2 = soft and shaped, score 3 = loose, and score 4 = watery, where scores of 1 and 2 represented normal feces and scores of 3 and 4 represented diarrhea. For each pen, a single observer assigned the relative proportion of visible feces that fell within each category, as well as an overall pen score (Perez-Palencia et al. 2021).

On days 10 and 20, a blood sample was taken from 3 pigs that were within the average weight of the pen for concentration of serum IgA (n = 27/dietary treatment). Plasma was collected by centrifugation (2,000 × *g*, 15 min, 4 °C), placed in 1.5 mL microcentrifuge tubes and stored at −20 °C until analysis (CR412, Jouan Inc., Winchester, VA). Plasma IgA was analyzed according to the method described by Chaytor et al. (2011) using commercially available kit (Pig IgA ELISA Kit, Bethyl Laboratories).

The differential sugar absorption test (**DSAT**) performed to assess intestinal permeability (Wijtten et al. 2011) was completed for 3 d to coincide with blood collection (days 9 to 11 and days 19 to 21) using 1 barrow per pen. The relevant pig was selected from the pigs used for blood sampling. On each day of the DSAT test, the pigs were randomly transferred to one of 9 individual cages (0.56 × 0.64 × 0.89 m^2^) with access to feed and water. Pigs were orally administered to a bolus containing 5% lactulose (**L**) and mannitol (**M**) at 15 mL/kg (Nguyen et al. 2014) using a syringe plus a liquid feeding tube followed by total urine collection for 6 h (Perez-Palencia et al. 2021). Thereafter, the pigs were transferred back to their original pen. A subsample of urine was collected after homogenization and stored at −80 °C for subsequent determination of lactulose–mannitol (**L:M**) ratio using commercially available kit (EnzyChrom Intestinal Permeability Assay Kit Catalog No: EIPM-100) as a marker of intestinal permeability (Hong et al. 2020).

### Apparent Total Tract Digestibility

Apparent total tract digestibility (**ATTD**) of dry matter (**DM**), crude protein (**CP**), gross energy (**GE**), and phosphorus of experimental diets were calculated according to the indirect evaluation method during Phase IV (days 25 to 35) using 0.4% celite as the indigestible marker (Kiarie et al. 2016). From days 32 to 35, fresh fecal samples were collected once a day from each experimental pen. The DM concentration in the diets was determined by drying samples at 102 °C for 24 h using a drying oven and ground to pass through a 0.5 mm screen using a mill grinder (Retsch zm 200, ring sieve size: 0.75 mm). The GE concentration in diets and fecal samples were analyzed by bomb calorimetry (Parr 6300 calorimeter, Parr Instruments Co., Moline, IL). The CP (method 990.03), crude fat (Ether Extraction, AOAC Official Method 920.39), crude fiber (CF, AOAC Official Method 978.10, 2006), neutral detergent fiber (NDF, JAOAC 56, 1352-1356, 1973), acid detergent fiber (ADF, AOAC Official Method 973.18 (A-D), 2006), and phosphorus (P, method AOAC Official Method 966.01) were determined at a commercial laboratory (University of Missouri, Columbia MO). To determine ATTD of nutrients and phosphorus, the following equation described by Adeola (2001) was used:

$Digestibility\;(\%)=100-\left[100\times \left(\frac{M_{feed}\times C_{feces}}{M_{feces}\times C_{feed}}\right)\right]$

where *M*_feed_ and *M*_feces_ represent concentrations of marker compound in feed and feces, respectively; *C*_feed_ and *C*_feces_ represent concentrations of marker in feed and feces, respectively.

### Statistical Analysis

The SAS UNIVARIATE procedure was used to confirm the homogeneity of variances and test for outliers. Data were analyzed as a randomized incomplete block design using the PROC MIXED procedure in SAS. In the model, dietary treatment was considered the main effect and room and BW as the blocking factor with pen as the experimental unit for performance measures. For serum and intestinal permeability assessments, individual pig was the experimental unit. Tukey’s adjusted means test was used to detect differences between treatment groups where main effect of treatment was significant. Significance was considered at *P* ≤ 0.05. For analysis of fecal scores, data were analyzed using the PROC FREQ procedure in SAS.

## RESULTS

### Diets Composition

The analyzed chemical composition of experimental diets is presented in Tables 3 and 4. Regarding one of the most limiting AA, the total Lys concentration was within 1.38% (SD = 0.08) for the 4 phases. For the second limiting AA, Thr concentration was within 0.83% (SD = 0.05) between diet phases, and the variation in Met concentration was within 0.39% (SD = 0.02) between diet phases. Within the analyzed branched chain amino acid (**BCAA**) values, the variation in Leu concentration was within 1.69% (SD = 0.09). While the variation Ile and Val concentration was 0.8% (SD = 0.02) and 0.91% (SD = 0.05), respectively, throughout all phases. The highest Leu to Lys ratio was in FM/ESBM in phase 1 and SBM75 in phase 2; when compared with CON, the SID Leu to Lys increased from 104% to 121%, and 114% to 131%, respectively. The highest Leu to Lys ratio was in SBM75 in phase 3 and FM/ESBM in phase 4; when compared with CON, the SID Leu to Lys increased from 114% to 138%, and 116% to 138%, respectively.

**Table 3.** Analyzed chemical composition (as-fed basis) of experimental diets (phases 1 and phase 2)^1^

Ingredient	Phase 1				Phase 2			
	CON	SBM75	FM/ESBM	GDDY50	CON	SBM75	FM/ESBM	GDDY50
Gross energy, kcal/kg	3,867	3,972	3,906	3,917	3,821	3,919	3,860	3,880
Crude fat, %	2.80	3.09	2.69	3.06	2.62	2.70	2.47	2.82
Crude fiber, %	1.53	2.03	2.23	1.95	1.61	2.10	2.09	2.10
Crude protein, %	21.0	21.3	20.3	19.5	19.8	20.4	19.4	19.7
*Total, %*								
Lys	1.55	1.70	1.73	1.68	1.49	1.35	1.51	1.69
Met	0.44	0.45	0.47	0.45	0.38	0.42	0.37	0.39
Met + Cys	0.79	0.81	0.88	0.83	0.68	0.80	0.74	0.74
Thr	1.01	1.09	1.03	0.98	0.87	0.91	0.90	0.89
Trp	0.23	0.23	0.24	0.25	0.22	0.22	0.23	0.23
Ile	0.98	0.97	1.04	0.94	0.90	0.94	0.94	0.91
Leu	1.70	1.87	1.98	1.80	1.69	1.86	1.77	1.77
Val	1.07	1.07	1.13	1.07	0.97	1.06	1.02	0.99
His	0.49	0.51	0.55	0.49	0.48	0.51	0.50	0.49
Phe	0.92	0.92	1.01	0.90	0.87	0.92	0.92	0.89
Ash	7.15	7.47	7.30	7.53	6.45	7.23	6.69	6.86
Phosphorus	0.63	0.67	0.64	0.65	0.59	0.61	0.60	0.61

^1^Dietary treatments: CON: standard nursery diet; SBM75: CON replacing 75% of SBM by GDDY; FM/ESBM: CON without fishmeal and ESBM + GDDY inclusion; HP-GDDY50: CON replacing 50% of SBM, FM, and ESBM by GDDY. Experimental diets were formulated in a 4-phase program with phase 1 from weaning to day 7, phase 2 from days 7 to 21, phase 3 from days 21 to 28, and phase 4 from days 28 to 53.

**Table 4.** Analyzed chemical composition (as-fed basis) of experimental diets (phase 3 and phase 4)^1^

Ingredient	Phase 3				Phase 4			
	CON	SBM75	FM/ESBM	GDDY50	CON	SBM75	FM/ESBM	GDDY50
Gross energy, kcal/kg	3,845	3,926	3,832	3,925	3,823	3,951	3,803	3,935
Crude fat, %	2.71	2.95	2.16	2.78	2.17	3.06	1.86	2.55
Crude fiber, %	1.85	2.45	2.02	2.19	2.03	2.87	2.00	2.45
Crude Protein, %	20.2	21.4	20.1	20.1	18.3	18.6	18.4	18.9
*Total, %*								
Lys	1.41	1.52	1.39	1.51	1.36	1.54	1.30	1.33
Met	0.38	0.39	0.42	0.38	0.35	0.35	0.34	0.37
Met + Cys	0.72	0.75	0.77	0.74	0.66	0.70	0.65	0.73
Thr	0.89	0.89	0.95	0.91	0.84	0.80	0.81	0.86
Trp	0.22	0.21	0.23	0.22	0.19	0.19	0.18	0.19
Ile	0.89	0.85	0.93	0.91	0.82	0.79	0.81	0.82
Leu	1.60	1.84	1.73	1.86	1.53	1.76	1.55	1.80
Val	0.97	0.99	1.00	1.04	0.88	0.94	0.88	0.96
His	0.48	0.50	0.52	0.53	0.48	0.49	0.48	0.51
Phe	0.91	0.90	0.98	0.95	0.90	0.89	0.90	0.93
Ash	6.36	6.76	6.38	6.59	5.43	6.12	5.60	5.76
Phosphorus	0.61	0.63	0.59	0.62	0.61	0.58	0.58	0.57

^1^Dietary treatments: CON: Standard nursery diet; SBM75: CON replacing 75% of SBM by GDDY; FM/ESBM: CON without Fishmeal and ESBM + GDDY inclusion; HP-GDDY50: CON replacing 50% of SBM, FM, and ESBM by GDDY. Experimental diets were formulated in a 4-phase program with phase 1 from weaning to day 7, phase 2 from days 7 to 21, phase 3 from days 21 to 28, and phase 4 from days 28 to 53.

### Growth Performance

During the first two periods (days 0 to 7 and days 7 to 14), no significant differences were observed in BW, ADG, and ADFI among treatment groups (Table 5). However, at day 21, pigs fed the CON diet had greater BW (*P* = 0.01) than those fed the GDDY50 diet, while ADG was higher (*P* = 0.000) in pigs fed CON diet compared with those fed SBM75 and GDDY50. During days 21 to 28 period, dietary treatments had no significant effect on BW, ADG, and ADFI; although CON-fed pigs tended (*P* = 0.092) to have a greater BW in comparison with pigs fed GDDY50. During days 28 to 42 period, pigs fed CON had grater BW, ADG, and ADFI (*P* < 0.0001) than those fed SBM75 and GDDY50. During days 42 to 53 period, pigs fed CON had greater BW than those fed SBM75, and GDDY50, while pigs fed CON had grater ADG and ADFI (*P* < 0.0001) than those fed SBM75. Pigs fed CON and FM/ESBM had greater gain to feed (G:F) ratio than SBM75-fed pigs during days 7 to 21, and all 3 diets had a higher G:F ratio than SBM75-fed pigs during days 28 to 53.

**Table 5.** Main effects of dietary corn-fermented protein product (GDDY) inclusion on pig growth performance throughout the nursery period^1^

Item^3^	Dietary treatments				SEM	*P*-value^2^
	CON	SBM75	FM/ESBM	GDDY50		
Initial BW, kg	5.66	5.67	5.68	5.64	0.03	0.851
*Period, days 0 to 7*						
BW day 7, kg	6.1	6.1	6.1	6.1	0.06	0.834
ADG, g	68	65	58	62	5.69	0.678
ADFI, g	103	93	100	97	4.25	0.451
F:G	1.56	1.44	1.73	1.60	0.09	0.169
G:F	0.70	0.76	0.65	0.70	0.05	0.423
*Period, days 7 to 14*						
BW day 14, kg	6.7	6.5	6.5	6.4	0.10	0.305
ADG, g	140	140	140	140	0.01	0.238
ADFI, g	220^x^	202^xy^	210^xy^	193^y^	7.25	0.075
F:G	1.78	1.96	1.96	2.02	0.20	0.828
G:F	0.61	0.58	0.56	0.57	0.04	0.806
*Period, days 14 to 21*						
BW day 21, kg	8.3^a^	7.9^ab^	8.1^ab^	7.7^b^	0.13	0.010
ADG, g	232^a^	190^bc^	230^ab^	179^c^	10.83	0.000
ADFI, g	353	345	355	315	10.51	0.040
F:G	1.63^a^	2.02^b^	1.78^ab^	1.87^ab^	0.08	0.030
G:F	0.65^a^	0.54^b^	0.63^ab^	0.57^ab^	0.03	0.040
*Period, days 21 to 28*						
BW day 28, kg	9.9^x^	9.5^xy^	9.8^xy^	9.4^y^	0.18	0.092
ADG, g	239	238	238	241	15.87	0.999
ADFI, g	419	391	394	388	15.21	0.430
F:G	1.75	1.74	1.68	1.67	0.11	0.918
G:F	0.57	0.57	0.57	0.57	0.04	0.806
*Period, days 28 to 42*						
BW day 42, kg	16.9^a^	14.2^b^	16.1^a^	14.9^b^	0.293	< 0.0001
ADG, g	500^a^	338^d^	448^b^	395^c^	10.58	< 0.0001
ADFI, g	749^a^	578^c^	687^ab^	627^bc^	18.11	< 0.0001
F:G	1.50^b^	1.72^a^	1.53^b^	1.59^b^	0.03	< 0.0001
G:F	0.67^a^	0.58^b^	0.66^a^	0.63^a^	0.01	< 0.0001
*Period, days 42 to 53*						
BW day 53, kg	24.4^a^	19.7^c^	23.3^ab^	21.4^bc^	0.52	< 0.0001
ADG, g	704^a^	524^b^	694^a^	613^ab^	25.38	< 0.0001
ADFI, g	1,164^a^	912^b^	1,150^a^	1,022^ab^	43.16	0.001
F:G	1.66	1.75	1.67	1.69	0.06	0.715
G:F	0.61	0.61	0.61	0.61	0.02	0.887
*Period, days 0 to 53*						
ADG, kg	360.1^a^	270.5^c^	339.1^ab^	302.8^bc^	9.84	< 0.0001
ADFI, kg	501.9^a^	420.5^c^	482.9^ab^	440.8^bc^	11.32	< 0.0001
F:G	1.41^b^	1.58^a^	1.43^b^	1.49^ab^	0.03	0.001
G:F	0.72^a^	0.64^b^	0.70^a^	0.69^ab^	0.01	0.004

^1^Dietary treatments: CON: Standard nursery diet; SBM75: CON replacing 75% of SBM by GDDY; FM/ESBM: CON without fishmeal and ESBM + GDDY inclusion; HP-GDDY50: CON replacing 50% of SBM, FM, and ESBM by GDDY. Experimental diets were formulated in a 4-phase program with Phase 1 from weaning to day 7, Phase 2 from days 7 to 21, phase 3 from days 21 to 28, and phase 4 from days 28 to 53.

^2^Superscripts ^abcd^ indicate significant difference at *P* ≤ 0.05 and ^wxyz^ indicate tendency at 0.05 < *P *≤ 0.10 using Tukey’s means separation test.

^3^ADFI, average daily feed intake; ADG, average daily gain; BW, body weight; G:F, gain to feed ratio; F:G, feed to gain ratio.

Considering the overall period (days 0 to 53), ADG and ADFI was greater (*P* < 0.0001) in pigs provided CON in comparison with SBM75- and GDDY50-fed pigs, while G:F ratio was greater in pigs fed CON and FM/ESBM when compared with SBM75.

### Fecal Score and Intestinal Health

**Figure 1.**
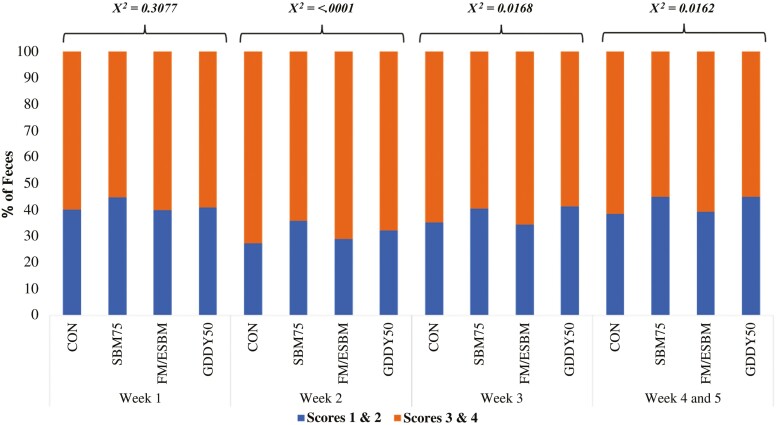
Relative proportion of pen fecal scores in nursery pigs provided diets containing varying proportions of corn-fermented protein product (GDDY).Dietary treatments: CON: Standard nursery diet; SBM75: CON replacing 75% of SBM by GDDY; FM/ESBM: CON without Fishmeal and ESBM + GDDY inclusion; GDDY50: CON replacing 50% of SBM, FM, and ESBM by GDDY. Experimental diets were formulated in a 4-phase program with Phase 1 from weaning to day 7, Phase 2 from days 7 to 21, phase 3 from days 21 to 28, and phase 4 from days 28 to 53.

Dietary treatments had no significant effect (*X*^*2*^ > 0.05) on pen fecal scores (Figure 1) during days 0 to 7; however, during days 7 to 35, pigs fed the diets with the greater inclusion of GDDY (SBM75 and GDDY50) had around 5% less incidence of watery and soft feces in comparison with pigs fed CON and FM/ESBM.

The DSAT and IgA data are presented in Table 6. On days 10 and 20, dietary treatments had no effect on urinary lactulose and mannitol concentrations or L:M ratio. However, urinary L:M ratio on day 20 tended to be greater (*P* < 0.099) in SBM75-fed pigs compared with pigs provided CON and GDDY50.

**Table 6.** Urinary lactulose and mannitol and serum Immunoglobulin-A in nursery pigs provided diets containing varying proportions of a corn-fermented protein product (GDDY)^1^

Item	Dietary treatments^1^				SEM	*P*-value^2^
	CON	SBM75	FM/ESBM	GDDY50		
*Day 10 postweaning*						
Lactulose, mM	0.054	0.076	0.057	0.081	0.0171	0.278
Mannitol, mM	0.113	0.158	0.100	0.101	0.0270	0.451
L:M	0.476	0.478	0.568	0.798	0.1244	0.330
IgA, mg/mL	0.162	0.154	0.168	0.171	0.0077	0.422
*Day 20 postweaning*						
Lactulose, mM	0.038	0.046	0.035	0.037	0.0067	0.683
Mannitol, mM	0.137	0.122	0.119	0.157	0.0334	0.857
L:M	0.274^z^	0.377^y^	0.292^z y^	0.232^z^	0.0365	0.099
IgA, mg/mL	0.192^yz^	0.175^z^	0.187^z^	0.209^y^	0.0094	0.082

^1^Dietary treatments: CON: Standard nursery diet; SBM75: CON replacing 75% of SBM by GDDY; FM/ESBM: CON without Fishmeal and ESBM + GDDY inclusion; HP-GDDY50: CON replacing 50% of SBM, FM, and ESBM by GDDY. Experimental diets were formulated in a 4-phase program with Phase 1 from weaning to day 7, Phase 2 from days 7 to 21, phase 3 from days 21 to 28, and phase 4 from days 28 to 53.

^2^Superscripts ^abcd^ indicate significant difference at *P* ≤ 0.05 and ^wxyz^ indicate tendency at 0.05 < *P* ≤ 0.10 using Tukey’s means separation test.

^3^IgA, immunoglobulin A; L:M, lactulose to mannitol ratio.

Dietary treatments had no significant effect on serum IgA concentration on day 10. However, on day 20, pigs from GDDY50 tended to have greater (*P* < 0.082) serum IgA concentration when compared with SBM75 and FM/ESBM.

### Apparent Total Tract Digestibility

There were no differences among dietary treatments for DM, CP, and GE digestibility (Table 7). The ATTD of crude fat was greater (*P* < 0.0001) in FM/ESBM in comparison with other dietary treatments, although the SBM75 had the lowest digestibility of crude fat. Digestibility of fiber components (CF, NDF, and ADF) was greater (*P* < 0.0001) in CON and FM/ESBM in comparison with SBM75 and GDDY50. Phosphorus digestibility did not differ in CON and FM/ESBM, while FM/ESBM had greater (*P* < 0.0001) digestibility values compared with SBM75 and GDDY50.

**Table 7.** Apparent total tract digestibility (ATTD) of weaned pig diets containing varying proportions of a corn-fermented protein product (GDDY)

Item^2^	Dietary treatments^1^				SEM	*P*-value^3^
	CON	SBM75	FM/ESBM	GDDY50		
DM	71.14	71.01	70.52	70.72	0.623	0.893
CP	50.21	49.83	48.42	51.34	0.895	0.166
C.Fat	82.66^b^	75.56^d^	85.67^a^	78.98^c^	0.541	< 0.0001
C.Fiber	70.74^a^	52.00^b^	72.09^a^	55.97^b^	1.388	< 0.0001
NDF	66.66^a^	59.45^b^	69.04^a^	58.02^b^	1.343	< 0.0001
ADF	66.50^a^	53.54^b^	67.35^a^	51.83^b^	1.797	< 0.0001
GE	18.17	17.69	17.06	16.83	0.497	0.256
P	76.64^ab^	73.29^c^	78.58^a^	74.78^bc^	0.602	< 0.0001

^1^Dietary treatments^:^ CON: Standard wean phase 1 feeding program; SBM75: diet 1 replacing 75% of SBM with GDDY; FM/ESBM: diet 1 with no protein sources (fish meal and ESBM), adding GDDY; GDDY50: diet 1 replacing 50% of each protein source (SBM, Fish meal, and ESBM), and adding GDDY.

^2^DM, dry matter; CP, crude protein; C. Fat, crude fat; C. Fiber, crude fiber; NDF, neutral detergent fiber; ADF, acid detergent fiber; GE, gross energy.

^3^Superscripts a, b, c, d indicate significant difference at *P* ≤ 0.05 and w, x, y, z indicate tendency at 0.05 < *P *≤ 0.10 using Tukey’s means separation test.

## DISCUSSION

Overall, this work indicates that corn fermented protein products, such as GDDY, can be successfully integrated into nursery pig diets. Growth performance of pigs fed GDDY-containing diets was similar to CON during the initial 2 wk of the postweaning period; although, by week 3, when the inclusion level exceeded 15%, performance was negatively affected. In this study, when GDDY inclusion levels exceeded 15%, the Leu to Lys ratio approached 140%.

In other nursery trials, when high protein dry distillers products were included in phase 3 diets up to 30% (Yang et al. 2018a) or 40% (Yang et al. 2018b), the Leu:Lys ratios ranged from 120% to 160%. Similar to this work, at high Leu:Lys ratios, growth performance of weaned pigs was reduced. The effect of high Leu:Lys ratio when main protein sources are replaced with GDDY or other corn-fermented protein products (Yang et al. 2018a, Cemin et al. 2021) has been well documented in growing-finishing pigs (Rojo 2011, Cemin et al. 2019, Clizer 2021, Kwon et al. 2022). It is suggested that in growing finishing pigs Leu:Lys ratios above 150% are expected to negatively impact performance (Htoo et al. 2014, Cemin et al. 2019, Kwon et al. 2019). Optimal SID Leu:Lys ratio in weaned pigs is suggested to be between 102% and 108% (Gloaguen et al. 2013 and Wessels et al. 2016). This work and Yang et al. (2018a, 2018b) suggest pigs in the latter nursery phase may be more susceptible to excess Leu:Lys ratios. Excess Leu generates an antagonistic effect between BCAA because they share the same enzyme complex (BCAAs aminotransferase and BCAA a-ketoacid dehydrogenase complex) as the first step in their degradative pathway (Langer et al. 2000, Sanderson and Naik 2000, Wiltafsky et al. 2010). Therefore, increasing the ratio of Ile and Val to Lys as the Leu:Lys ratio increases can be used to avoid detrimental impacts on growth performance (Htoo et al. 2007, Cemin et al. 2021). Acosta et al. (2021) suggested including up to 10% of corn-fermented protein products in diets for weaned pigs during phase 1 and 2 to reduce the negative effects on growth performance caused by the high concentration of Leu, which may negatively impact metabolism of Val and Ile.

It should be noted that in the first 3 wk after weaning pig BW and growth were slightly lower than expected (when compared to previous groups in the facility). It is expected this was because the pigs used in this study were weaned 3 days earlier (18.5 days) than the target 21 days of age due to a change in the facility’s weaning cycle. The overall poor performance could be attributed to the weaning age (Jang, et al. 2021). In this study, pig age at weaning was considered as a randomization criterion for pig distribution to pens. Thus, all experimental units had similar average weaning age and, therefore, the influence of this factor on pig growth responses was the same across dietary treatments.

One of the responses of the pig’s immune system to protect the intestinal epithelium from pathogens and toxins is intestinal production of IgA, which acts as a first line of defense through receptor blockade (Mantis et al. 2011). Therefore, serum IgA concentration was measured to assess gut health (Peng et al. 2021). During this study, the higher IgA concentration in pigs fed GDDY50 at day 20, compared to pigs fed SBM75 indicates a potential reduction in the immune response with partial replacement of SBM and high quality proteins (FM, ESBM) by GDDY. This lower immune response suggests a reduced ability of the immune system to effectively defend the body against pathogens.

An increase in intestinal permeability in weaned piglets begins around 24 h after weaning and recovery of the increased permeability occurs gradually over time, generally after week 2 in the nursery (Moeser et al. 2007). The DSAT test measures the ability of two unmetabolized sugar molecules, lactulose and mannitol, to permeate the intestinal mucosa and in this way evaluate intestinal permeability (Wijtten et al. 2011). Due to its relatively larger size compared to other molecules such as mannitol, lactulose enters the bloodstream through the paracellular route or via damage to the intestinal epithelium at the tight junction barrier that allows greater penetration of large molecules (Vojdani 2013). Therefore, an increase in the L:M ratio indicates lesser intestinal barrier function. The observed higher L:M ratio in SBM75 and FM/ESBM-fed pigs compared to CON-fed pigs implies that diet plays a role in altering small intestinal permeability, possibly enabling the passage of larger molecules, bacteria, and toxins into the bloodstream. However, incorporating GDDY at levels lower than 15% may help to mitigate these detrimental impacts on intestinal health. There was a tendency, especially at day 20 postweaning, for the urinary L:M ratio in SBM75 to be higher, which may indicate that the SBM75 diet has a negative effect on the intestinal barrier, which is also related with the lower immune response observed. In addition, the negative effect on gut health could result in a higher incidence of loose feces, however, higher occurrence of loose feces, up to 70%, was observed in pigs fed diets containing SBM instead of GDDY, which may be attributed to antinutritional factors in SBM that can cause temporary hypersensitive diarrhea (Liying et al. 2003, Kim et al. 2015). Diarrhea may be ascribed to an imbalance in intestinal absorptive and secretory function resulting in hypersecretion of ions and migration of water into the intestinal lumen driven by osmosis (Girard and Bee 2020). This sequence of events may be due, among other things, to the ingredient’s composition present in diets, these factors, along with NSPs in the ingredients, can alter the intestinal microflora and affect physiological functions, such as digestion transit times and mucosal cell turnover rates (Kiarie et al. 2013). Postweaning diarrhea can be exacerbated by the immaturity of the immune and digestive systems during the initial weaning phase (Lallès et al. 2007). Overall, the findings suggest that substitution of SBM with GDDY in pig diets may contribute to lower intestinal barrier immunological activity, alter intestinal barrier, and reduce the incidence of loose feces.

The ATTD of GE can be negatively affected as dietary fiber increases (Wang et al. 2016, Li and Patience 2017). However, in this study, the diets containing GDDY had up to 2.87% greater crude fiber levels when GDDY was included at greater than 14% without negatively affecting ATTD of GE. There was no difference in ATTD of CP between dietary treatments, which may suggest that there was no negative impact related to the proteolytic enzymatic digestion of CP (Morel et al. 2006). The ATTD of P was higher in the experimental diets in comparison with the ATTD of P in DDGS (60%) and HP-DDG (64%) reported by NRC (2012), which may be related to the fermentation process to obtain GDDY. During fermentation some of the phytate bound P is hydrolyzed, therefore, more P is available for absorption in the small intestine of the pig (Widmer et al. 2007). Consequently, the utilization of organic P is increased, and the need for supplemental inorganic P is reduced if ingredients with higher P digestibility are included in formulations at the expense of corn (Pedersen et al. 2007). However, in this study, this utilization of organic P was not estimated during the formulation of the experimental diets, which could explain the digestibility values for the diets containing GDDY. The ATTD of CF, NDF, and ADF was lower in SBM75 and GDDY50 diets, which is likely due to the higher dietary fiber content in these diets compared with CON and FM/ESBM diets, as fiber digestibility is susceptible to hindgut development conditions and a major content fiber in diet reduces nutrient digestibility (Zhao et al. 2018, Lyu et al. 2019).

## CONCLUSIONS

The study indicates that GDDY can be included up to 14% during the first 2 wk after weaning without compromising growth performance and that GDDY can partially replace SBM and completely replace high-quality proteins (FM and ESBM) in the first 2 wk after weaning. The inclusion of GDDY as a replacement for SBM reduced the incidence of diarrhea in the first weeks which may contribute to improved overall health status after weaning. In the later nursery period, it is important to consider the GDDY inclusion level to maintain a suitable balance of BCAA where higher levels of dietary Leu may compromise pig growth performance. Overall, GDDY is a valuable feedstuff to be included in nursery pig diets, especially during the first couple weeks after weaning. Strategies to minimize the effect of high BCAA concentration in GDDY are necessary to increase its inclusion in later nursery diets.
